# Quantum measurement enables single biomarker sensitivity in flow cytometry

**DOI:** 10.1038/s41598-023-49145-7

**Published:** 2024-02-16

**Authors:** J. Sabines-Chesterking, I. A. Burenkov, S. V. Polyakov

**Affiliations:** 1 Joint Quantum Institute, University of Maryland, College Park, 20742 USA; 2 National Institute of Standards and Technology, Gaithersburg, MD 20899 USA; 3 Department of Physics, University of Maryland, College Park, 20742 USA

**Keywords:** Single photons and quantum effects, Flow cytometry, Optical sensors, Quantum optics

## Abstract

We present the first unambiguous experimental method enabling single-fluorophore sensitivity in a flow cytometer using quantum properties of single-photon emitters. We use a quantum measurement based on the second-order coherence function to prove that the optical signal is produced by individual biomarkers traversing the interrogation volume of the flow cytometer from the first principles. This observation enables the use of the quantum toolbox for rapid detection, enumeration, and sorting of single fluorophores in large cell populations as well as a ‘photons-to-moles’ calibration of this measurement modality.

## Introduction

Flow cytometry is a powerful technique for performing a wide range of measurements on cells in a high-throughput fluorescence-based multi-parametric analysis and cell-by-cell sorting^1^. The use of fluorescent biomarkers in flow cytometry enables the detection of specific target cells or biomolecules in inhomogeneous populations allowing for applications such as medical diagnosis^2–4^ or drug discovery^5^. On the other hand, single fluorescent molecule detection and counting methods are mainly limited to low-throughput measurements such as single-molecule microscopy imaging where target molecules are immobilized. This is because conventional flow cytometers lack desired sensitivity to detect the signal from a single fluorescent biomarker. We use optical quantum measurements to characterize the photo-emission of individual biomarkers and set up a cut-off-based identification of single-emitters vs. classical detection events in flow cytometer settings.

The objective of the method presented in this work is to identify biomarkers that are associated with rare events, such as cells comprising less than 0.01% of a population^6,7^ or precisely quantifying the number of proteins or genes present in a cell^8–10^. However, the low photoemission rate of individual biomarkers, combined with detector background noise, variations in laser intensity, Raman scattering, and contaminants in the flow, make detecting single-emitters a challenging task^11–14^.

Here, to achieve and verify the desired sensitivity, we implement a measurement method that employs the quantum properties of single biomarkers. By performing a second-order coherence measurement ($g^{(2)}(0)$) using a Hanbury Brown and Twiss (HBT) setup^15^, we can unambiguously determine if the source of our signal is due to single-emitters in the interrogation volume, guaranteed by the laws of quantum mechanics^16^. We tested our method with a highly diluted concentration of quantum dots and obtained a value of $g^{(2)}(0)=0.20(14)$, this implies that the detected signal was predominantly generated by individual emitters^17–19^. (The digit(s) in parentheses are the uncertainty, to the precision of the same number of least significant digit(s) corresponding to one standard deviation.) The observation of antibunching (i.e. $g^{(2)}(0)<1$) is required to prove the single-emitter character of detected light^15,16,20^. Single fluorophore detection was achieved in static liquid solutions^18,21–24^, yet no such measurement has been reported to date in a flow cytometer where every fluorophore is interrogated only once and for a very short period of time. The non-classical HBT measurements are not reported even in more recent publications^22,23^ where the innovative technique of two-color coincident detection in conjunction with labeling the target molecule with two different fluorophores was introduced. We point out that in addition to providing proof of single fluorophore sensitivity the HBT measurement is required for the absolute quantification of the number of detected fluorophores, e.g. for distinguishing fluorescent activity from one vs. a few emitters, leading to new practical applications in life sciences and medicine.

In addition to being the first instance of single-emitter resolution in flow cytometry verified by a quantum measurement, this method enables bounding the single-emitter signal-to-noise ratio (SSNR) and discriminating one and few-emitter activity from classical bursts of light from first principles of quantum mechanics. It also enables absolute flow cytometer calibration, absolute concentration measurements^25^, and accurate molecule counting^20^. We anticipate that the ability to resolve the number of emitters can find multiple applications, for instance in cancer diagnosis^26,27^ and gene editing with CRISPR^28,29^ where identifying the exact number of copies of a gene is an invaluable tool. The high value of SSNR experimentally measured in this work validates the feasibility of HBT measurements in flow cytometry settings and enables applications, that we have theoretically described in Ref.^25^. Moreover, our setup can be used to analyze the stoichiometry of molecules marked with different number of fluorophores^30^.

## Results

**Figure 1:**
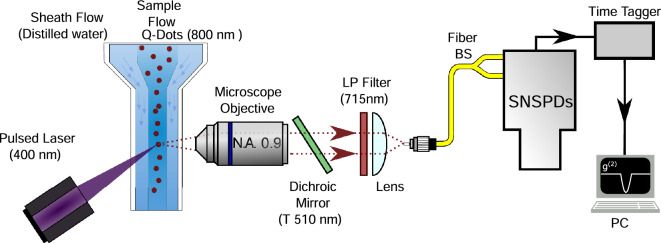
Experimental setup. Two syringe pushers independently control the sample flow with quantum dots (QD) and the sheath flow (distilled water) through a $0.25\times 0.25$ mm flow channel. Picosecond pulses at 405 nm with a repetition rate of 76 MHz excite quantum dots in the flow channel. Quantum dots fluoresce at $\approx 800$ nm. An objective with 0.9 numerical aperture (NA) collects fluorescent light orthogonally to both the direction of the flow and the pump laser beam. After filtering by a dichroic mirror (low pass at 510 nm) and a thin film filter (low pass at 715 nm), fluorescent photons are coupled into a single-mode fiber. A 50:50 fiber beam splitter sends the output from the flow cell to two superconducting nanowire single-photon detectors (SNSPD). A time tagger and a PC record and store photon arrival times for statistical processing.

The experimental setup of the home-built quantum flow cytometer is shown in Fig. 1. It consists of a flow cell, an excitation source, and a pair of superconducting nanowire single photon detectors (SNSPD) in the HBT configuration^15^ that analyze the fluorescence from the sample coupled in the single-mode fiber. Time of arrival information of the detection events is obtained using a time-tagger and a personal computer is used to record photon arrival times from the two detectors for statistical processing, including signal-to-noise optimization.

The excitation source is a picosecond pulsed Ti:sapphire laser frequency-doubled to $\approx 405$ nm with a mean optical power of $\approx 100$ mW and repetition rate $r=76$ MHz. The sample is a highly-diluted suspension of commercially available CdSe colloidal quantum dots, QDs (Qdot 800 Streptavidin Conjugate from Thermo Fisher Scientific) with emission centered at $\approx 800$ nm in a phosphate-buffered saline (PBS) solution. (Note that certain commercial products or company names are identified in this manuscript to describe our study adequately. Such identification is not intended to imply recommendation or endorsement by the National Institute of Standards and Technology, nor is it intended to imply that the products or names identified are necessarily the best available for the purpose.) The large spectral detuning between the excitation and the fluorescent emission wavelengths (800 nm) is chosen to reduce the background from Raman scattering in water. A high numerical aperture (NA = 0.9) dry objective collects the fluorescent light from a direction orthogonal to the pump beam to reduce cross-talk from the laser. The collected light is filtered in two stages. First, a dichroic mirror rejects scattered pump light by reflecting wavelengths shorter than 510 nm. Second, a low-pass filter at 715 nm rejects light produced by the Raman scattering in water and further reduces scattered light from the pump. The filtered fluorescent signal is coupled into a single-mode fiber and directed into an HBT setup comprised of a 50/50 fiber beam splitter (FBS) and a pair of SNSPDs. Because our detectors are optimized for telecom band, measured detection efficiencies are very low at 800 nm: detection efficiencies of 0.035 and 0.04 were measured against a stock power meter. However, HBT measurements are insensitive to loss and therefore do not require a precise calibration of light collection and detection efficiencies in the experimental setup.

A commercial glass flow cell with a cross-sectional channel measuring 250 $\upmu$m $\times$ 250 $\upmu$m was employed. To ensure alignment of the sample within the center of the flow cell, the hydrodynamic focusing of the sample was achieved with distilled water as the sheath flow. Flow rates of 1 $\upmu$l/min and 5 $\upmu$l/min for sample and sheath flows respectively were set and controlled with syringe pushers.

To obtain a sample with a low enough concentration to ensure that we observe one or just a few biomarkers at a time, we estimated the optical interrogation volume (OIV) using the full width at half maximum (FWHM) of the axial and lateral optical beam distributions near the focal point for the collection lens^31^. Note that the focal spot of the excitation laser is significantly larger than that of the collection high NA objective, due to the flow cell design, which has a thinner glass layer on the collection side to allow for use of a high numerical aperture lens. The estimated OIV is of the order of 1 femtoliter. We use the nominal concentration of the commercial quantum dots provided by the manufacturer to assure the average number of quantum dots flowing through the OIV remained below one. Two samples with an average of $\approx 0.1$ and $\approx 0.5$ emitters in the OIV were prepared. We will refer to these samples as low concentration (LC) and high concentration (HC) respectively.

In our experiment, we show that cut-off detection enables recognizing bright events due to classical particles from one (or few) fluorescent emitters. Using the quantum second-order correlation function $g^{(2)}(0)$ we further show that we reach a single-emitter-level sensitivity and set a lower bound on the SSNR from first principles (see “Methods” for details).

We obtain a record of photon detections using an HBT setup from our flow cell with two different concentrations of QDs in a sample. We apply a $\approx 2.5$ ns temporal window for calculating $g^{(2)}(0)$ dependence on the photon count rate (PCR) cut-off. The temporal window is synchronized with the optical pump pulses such that most of the fluorescent signal including its peak is used. Detections outside of this window are discarded. In that way, the effect of the uncorrelated background on $g^{(2)}(0)$ is reduced (and SSNR is increased). We point out that this step is only possible with pulsed excitation and it significantly improves SSNR, see “Methods” for details.

**Figure 2:**
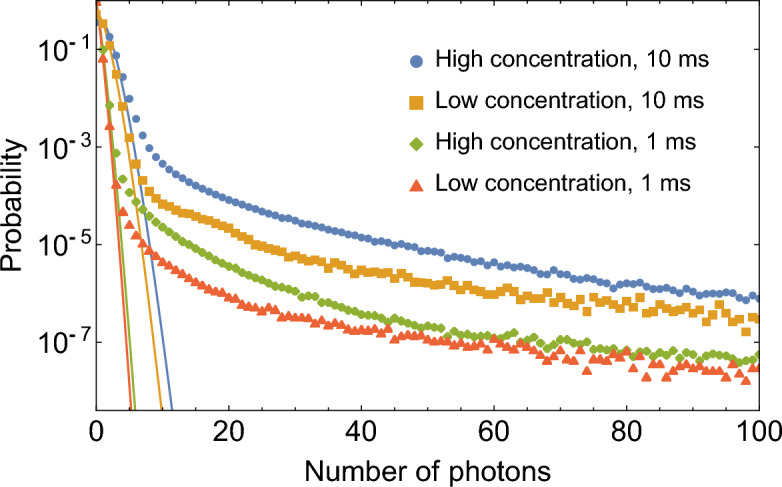
Photon number distributions $\wp (n)$ for high and low concentration measurements obtained using 1 ms and 10 ms time bins as labeled. Solid lines show Poisson fits of the photon number distributions.

To perform our analysis, we split the recorded number of photo-detection events in intervals $\tau$ of 1 ms and 10 ms, which corresponds to approximately 2 and 20 traversal times of a point size particle crossing the interrogation volume. Both 1 ms and 10 ms time bins are much longer than the pump laser repetition period of $1/r\approx 13.16$ ns and therefore include a large number of trials. For each interval we found the number of detected photons *n* and calculated photon-number distributions (PNDs) $\wp (n)$. Four PNDs (two for each low concentration and high concentration data sets) are shown in Fig. 2.

All PNDs exhibit a nearly Poisson distribution at low photon number values, and significantly deviate from Poisson statistics at higher photon numbers. This behavior is consistent with the flow of isolated quantum dots (described by the Poisson-like part of the PND) interspersed with rare classical particles (described by the long tails), see Fig. 2. It turns out that colloidal quantum dots tend to aggregate in polar solvents such as water and can form clusters^32–34^, which could be one of the causes for such classical bursts of light. Other possible causes could include residual impurities and air bubbles.

From Fig. 2, we observe that such bright events are extremely rare—they occur in fewer than 0.07% in 1 ms bins and fewer then 0.4% in 10 ms bins. Altogether, they account for $<0.2$% and $\lesssim 3$% of photons detected in the experiment for 1 ms bins and 10 ms bins, respectively. Yet, as it can be seen, these events contribute a significant number of coincidence events in the HBT analysis due to large *n*. In order to discard these rare but “bright” events, one can set an appropriate cut-off value $n_{\text {cut-off}}$ and truncate summations. For details, see Eq. (5) in “Methods”. Note that both 1 ms and 10 ms time intervals are only used to determine appropriate cut-offs (and reject data above the cut-off). Beyond rejection of data, the bin duration has no effect on $g^{(2)}(0)$ calculation.

**Figure 3:**
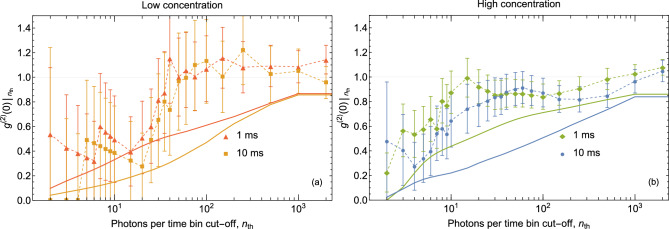
Second-order coherence function at zero time delay $g^{(2)}(0)$ as a function of the number of photons per time bin filter cut-off $n_\text {cut-off}$. (**a**) Low concentration sample. (**b**) High concentration sample. Error bars are statistical uncertainties calculated as the normalized square root of number of measured coincidences. A temporal filter is set to discard photons detected with delays greater than $\approx 2.5$ ns. Thin dashed lines—guides for an eye that connect experimentally obtained data points. Solid lines—$g^{(2)}(0)$ values from the model, see text.

Figure 3 shows $g^{(2)}(0)$ as a function of the number of photons per time bin filter cut-off $n_\text {cut-off}$ of detected photons for the two time intervals. The dependence of $g^{(2)}(0)$ on the number of photons per time bin filter cut-off is similar for all concentrations and intervals. Because of the low dark count rates of the SNSPD detectors, we present all results with no correction for dark counts.

## Discussion

Quantum measurements offer an independent, absolute characterization methods from the first principles. They can be applied here to verify the single-biomarker character of the emission and bound the SSNR from below. To this end, $g^{(2)}(0)<1$ is the evidence of non-classical light and $g^{(2)}(0)<0.5$ is a sign of emission predominantly from a single biomarker. Thus, we see statistical evidence of emission from predominantly one fluorescent biomarker at the time in all experiments and independently of the time intervals $\tau$ used.

To apply this method in practice, a theoretical model that ties the observed PCR to the properties of the sample is required. Remarkably, a simple model with no fitting parameters explains all the experimentally observed trends in $g^{(2)}(0)|_{n_{\text {cut-off}}}$ as a function of $n_\text {cut-off}$ extremely well (see Methods and refer to Eq. (5). The model also reveals that the experimental values of $g^{(2)}(0)$ are larger than that predicted by approximately 0.2. Because the model considers the ideal case, the values of $g^{(2)}(0)$ predicted by the model are expected to be lower than those obtained in the experiment. This important result shows that (a) single fluorescent biomarkers can be resolved in flow cytometry settings and (b) the PCR cut-off can be used to separate the detection of quantum particles from occasional classical bursts due to contaminating particles (such as QD clusters or air bubbles).

**Figure 4:**
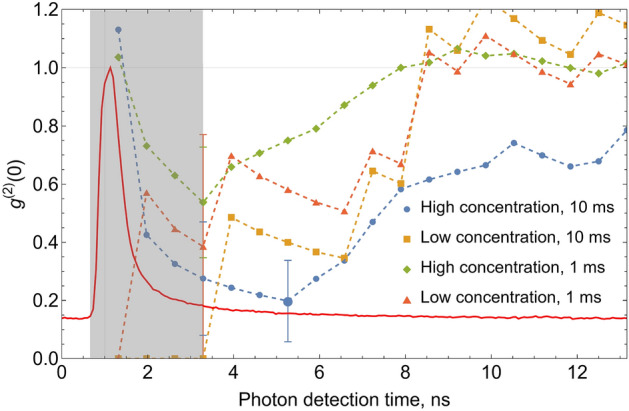
Temporal dependence of fluorescence and its use for filtering of the experimental data. Solid plot markers: The experimental values of $g^{(2)}(0)$ in the interval that begins at the beginning of the shaded region (0.65 ns) and ends at the time indicated by the *x* axis with a PCR cut-off set to 4 photons/bin. Dashed lines: guides for an eye connecting experimental points obtained for different concentrations and time bin sizes. Shaded region: temporal filtering of $\tau \approx 2.6$ ns used throughout the manuscript. Error bars are statistical uncertainties calculated as the normalized square root of number of measured coincidences and shown only for the points used in the manuscript. Large blue dot marks the lowest value of the second-order correlation function observed with a temporal window of 4.6 ns. Red solid line: observed fluorescence signal temporal profile (normalized by the maximum value).

Independent of the above results, the observed values of $g^{(2)}(0)$ set the lower bound for SSNR, see^25^. Particularly, the lowest value of the second-order correlation function observed with a temporal window of 4.6 ns is $g^{(2)}(0)\approx 0.20(14)$ (marked with a large blue point in Fig. 4), which corresponds to SSNR$\ge 6$ (where the upper bound is calculated using a conservative value for $g^{(2)}(0)$ obtained by adding one standard deviation to its measured value). This result verifies the single fluorophore sensitivity in a flow cytometry setting from the first principles. With improved detection efficiency our approach can be applied to a wide range of real-life problems from gene editing verification for quantitative medicine to rare-event detection for cancer diagnostics and post-cancer monitoring. This achievement opens the door for absolute calibration and cross-laboratory verification of the flow cytometer measurements, aiding to the proliferation of quantitative medicine.

## Methods

### Correlation measurements

Our proposed method takes advantage of the fact that fluorescent molecules and colloidal quantum dots (QD) commonly used as biomarkers in flow cytometry are single-photon sources. An ideal single-photon source cannot produce more than one photon at a time and requires some time to return to its emitting state. Therefore in the HBT^17^ optical setup consisting of a beamsplitter and a pair of single-photon detectors, the detectors theoretically will never click simultaneously. This phenomenon is known as antibunching and it can be characterized by a second-order coherence function $g^{(2)}(\Delta t)$ :

1 $$\begin{aligned} g^{\left( 2\right) }(\Delta t)=\frac{\left\langle {\hat{a}}^\dag \left( t\right) {\hat{a}}^\dag \left( t+\Delta t\right) {\hat{a}}\left( t+\Delta t\right) {\hat{a}}(t)\right\rangle }{\left\langle {\hat{a}}^\dag \left( t\right) {\hat{a}}(t)\right\rangle ^2}, \end{aligned}$$

where ${\hat{a}}^\dag$, ${\hat{a}}$ are creation and annihilation operators of the electromagnetic field^35^. In case of a zero delay $\Delta t=0$, Eq. (1) becomes:

2 $$\begin{aligned} g^{\left( 2\right) }(0)=\frac{\left\langle {\hat{n}}(t)({\hat{n}}(t)-1))\right\rangle }{\left\langle {\hat{n}}(t)\right\rangle ^2}, \end{aligned}$$

where ${\hat{n}}$ are photon number operators. Clearly, for the ideal single-photon emitter (${\hat{n}}(t)\in 0,1$), Eq. (2) gives zero. More generally, it can be shown that

3 $$\begin{aligned} g^{\left( 2\right) }(0)=\frac{(N-1)N}{N^2}<1, \end{aligned}$$

where *N* is the number of quantum emitters. This dependence of $g^{(2)}(0)$ on the number of emitters is independent on overall optical loss and is particularly strong for low *N*^36–40^. This anticorrelation (or antibunching) occurs with one or few emitters in contrast to a classical field, when $g^{\left( 2\right) }(0)\ge 1$. In practice, there is always a probability to obtain a false photodetection either because of dark noise of the detector or background noise due to a strong optical pump used to excite fluorophores. This noise is uncorrelated ($g^{\left( 2\right) }(0)=1$), and therefore it can be accounted for in the quantum measurement. If the probability to detect a photon from one single-photon emitter biomarker is $p_\text {b}$ and the probability to detect a noise photon is $p_\text {n}$ then SSNR = $p_\text {b}/p_\text {n}$. In our experiment the number of emitters that are present in the interrogation volume is governed by a Poisson process, the $g^{(2)}(0)$ depends on concentration (the average number of emitters in the measurement volume) $\langle N\rangle$:

4 $$\begin{aligned} g^{(2)}(0)|_{\langle N\rangle }\approx \left[ 1+\frac{\langle N\rangle \textrm{SSNR}^2}{(\langle N\rangle \textrm{SSNR}+1)^2}\right] ^{-1}. \end{aligned}$$

here, $g^{(2)}$ is normalized using measured correlation values in the immediate vicinity of $\Delta t = 0$^25^. Thus, remarkably, the non-classical $g^{(2)}(0)<1$ can be observed and fundamental features of $g^{(2)}(0)$ due to the quantum character of emitters can be extended and used in applications where the number of fluorophores in the interrogation volume fluctuates throughout the measurement, which is the case in flow cytometry.

Because, in practice, a fluorescent biomarker can sometimes emit more than one photon during the cycle and because the exact concentration $\langle N\rangle$ can be unknown, Eq. (4) bounds SSNR from below. Because antibunching cannot improve due to any competing classical light emission process, the analysis of this non-classical signature aids in SSNR optimization. In addition, it can be used to separate classical, quantum emission events, as discussed in this paper, even when attributes of classical, quantum fields (such as wavelength and lifetime) are the same.

### **Cut-off** identification of classical and quantum particles

In practice, our typical experiment yields a certain fraction of extremely bright events, which are due to classical-light emitting particles. In our experiment such events may come from large clusters of quantum dots or other fluorescing impurities. To distinguish between such classical events and the single-emitters we seek, we have established a photon count rate cut-off. To implement this cut-off, we have split the continuous record event dataset into intervals $\tau$ during which a total number of detected photons *n* is calculated. *n* is a random value, whose probability distribution $\wp (n)$ can be experimentally established and is referred to as a photon number probability distribution (PND). Using the number of detections in a particular time bin (the photon count rate), we can determine the likelihood $P_c(n)$ that the particle that caused this outcome is classical. Note that $1-P_c(n)$ yields the likelihood that the same outcome is due to a quantum event. Because the traversal time through the optical interrogation volume of single-emitters and classical-light emitting particles is the same, a classical correlation measurement cannot differentiate between those classical and quantum events, even in principle (c.f.^18,21–23^).

The fundamental laws of quantum optics can be used to verify this approach from first principles. In the following, we consider an HBT setup with two detectors with equal detection efficiencies. For simplicity, we assume that all quantum events are due to ideal single-emitters ($g^{(2)}(0)=0$), and all classical particles yield $g^{(2)}(0)=1$. We further assume low probability to detect a single photon per one excitation pulse. The average number of coincidences with a zero delay recorded during the observation interval $\tau$ is:

$$\begin{aligned} c_0(n) =\frac{\beta n^2}{\tau r} P_c(n), \end{aligned}$$

where $\beta =T_{\textrm{trav}}/\tau$ and $T_{\textrm{trav}}$ is the traversal time of a classical emitter. Here we do not expect more than one bright event during the observation interval $\tau$ because probability of such events is small (c.f. the tail of the experimental PND in Fig. 2). *r* is the pulse rate of the laser. At the same time, the average number of accidental coincidences is:

$$c_{a} (n) = \frac{{n^{2} }}{{\tau r}}\left( {1 - P_{c} (n)} \right) + \frac{{\beta n^{2} }}{{\tau r}}P_{c} (n)$$

As evident from the last two expressions, in this model, light from quantum particles does not contribute to coincidence detections *c* during the same excitation pulse, but contributes to random coincidences, where the two-photon detections occur due to two different excitation pulses, i.e. not quantumly correlated. Then, $g^{(2)}(0)$ can be found from:

5 $$\left. {g^{{(2)}} (0)} \right|_{{n_{{{\text{cut-off}}}} }} = \frac{{\sum\nolimits_{{n = 0}}^{{n_{{{\text{cut-off}}}} }} {\wp (n)c_{0} (n)} }}{{\sum\nolimits_{{n = 0}}^{{n_{{{\text{cut-off}}}} }} {\wp (n)c_{a} (n)} }},$$

Remarkably, all the parameters for this model are readily available in the experimental record, and no fitting is required. The PCR cut-off ($n_{\text {cut-off}}$) can be treated as a variable. We point out that this simple model assumes ideal, single sources of photons, and does not account for background and statistical effects due to the particle flow in a flow cell. Owing to the properties of $g^{(2)}$, these effects would increase the value of $g^{(2)}(0)$.

In order to apply this model to the experimental results, we first identify a part of the experimentally observed PND $\wp (n)$ that is described well by the Poisson distribution using non-linear fitting. Then we subtract the Poisson fit from the experimental PND and attribute positive residuals to rare classical events:

$$\begin{aligned} P_\text {c}(n)={\left\{ \begin{array}{ll}\frac{\wp (n) - \wp _\text {Poisson Fit}(n)}{\wp (n)} &{} \wp (n) > \wp _\text {Poisson Fit}(n)\\ 0 &{} \wp (n) < \wp _\text {Poisson Fit}(n) \end{array}\right. }. \end{aligned}$$

The traversal time for the model can be found from second-order correlation measurements by removing the cut-off ($n_{\text {cut-off}}=\infty$). The experimentally extracted $T_{\textrm{trav}}=0.47(1)$ ms, where uncertainty is one standard deviation of the Gaussian fit to a $g^{(2)}$ envelope (not shown). This value is close to the estimation of 0.31 ms obtained by dividing the focal diameter of the interrogation volume by the flow rate.

For the analysis of the experimental data, namely the measured $g^{(2)}(0)$ dependence on the PCR cut-off, we analyze detection time windows using $\tau$ of 1 ms and 10 ms. If the number of detections is above the cut-off, the segment is discarded. If that value is below the cut-off $n_{\text {cut-off}}$, we calculate the number of coincidences $c_0$ and the number of accidental coincidences $c_a(m)$ within the time window. The number of accidental coincidences is obtained by counting the number of two-detection events when the second detector fires exactly *m* laser excitation pulses after the first one. In our measurement, we average $c_a(m)$ over *m* using $m=2,3,\ldots ,250$, which reduces the statistical uncertainty of $c_a$ by $\approx 16$ times. In this way, the statistical uncertainty in calculating $g^{(2)}(0)$ is mainly determined by that of $c_0$.

### Temporal filtering of the fluorescent signal

One of the distinguishing features of our flow cytometer experimental setup is pulsed pump laser source. Short sub-picosecond laser pulses with repetition rate of 76 MHz allow HBT measurement synchronization and enable temporal filtering of the collected optical signals. We present the dependence of $g^{(2)}(0)$ on the width of the temporal window used to distinguish fluorescence light from background with a photon count rate (PCR) cut-off set to 4 photons/bin, Fig. 4. We find that the best results are observed when the temporal window begins at the point where the fluorescence peak just becomes resolvable from the background. In all cases, the observed $g^{(2)}(0)$ values grow with the window length due to optical background. Because the measurement is statistical, extremely short windows exhibit significantly larger uncertainties than longer ones. The optimal window duration that balances the two effects is $\approx 2.5$ ns. Temporal filtering of the fluorescent signals can be applied to fluorophores with longer lifetimes. In this case, the interval between laser pulses and the temporal window can be made longer and the overall signal-to-noise figure can be kept the same or similar (assuming no dark counts on detectors). Because SNSPDs have low dark count rates, this assumption should be reasonable even for fluorophores with significantly longer lifetimes. Note that the data presented in this work is not corrected for dark counts of the detectors.

The lowest observed value of $g^{(2)}(0)$ in this experiment is 0.20(14). It corresponds to a temporal filtering of $\approx 4.6$ ns for high concentration data, using 10 ms bin and maximum PCR filter set to 4 photons/bin. We point out that this result is nearly the same within the statistical accuracy as $g^{(2)}(0)=0.28(19)$ observed with the $\approx 2.5$ ns temporal window used through the manuscript.

## Data Availability

The datasets used and/or analysed during the current study available from the corresponding author on reasonable request.
